# Common Mental Disorders and Risk Factors in Urban Tanzania

**DOI:** 10.3390/ijerph7062543

**Published:** 2010-06-14

**Authors:** Rachel Jenkins, Joseph Mbatia, Nicola Singleton, Bethany White

**Affiliations:** 1 WHO Collaborating Centre (Mental Health), Institute of Psychiatry, Kings College London, UK; E-Mail: Rachel.jenkins@kcl.ac.uk; 2 Mental Health, Ministry of Health, Tanzania; E-Mail: jmbatia@muchs.ac.tz; 3 Policy & Research, UK Drug Policy Commission; UK; E-Mail: NSingleton@ukdpc.org.uk; 4 WHO Collaborating Centre (Mental Health), Institute of Psychiatry, Kings College London, UK; E-Mail: bwhite@nchecr.unsw.edu.au

**Keywords:** common mental disorder, Tanzania, poverty

## Abstract

A cross sectional population based epidemiological survey of 899 adults aged between 15 and 59 was undertaken in two urban areas of demographic surveillance sites in Dar es Salaam, Tanzania, using the Clinical Interview Schedule Revised. Significantly higher rates were found among those who had experienced more than three severe life events in the last six months and relationship difficulties and death of a loved one.

## 1. Introduction

Although most developing countries are expected to achieve the Millennium Development Goal of a 50% reduction in the number of people living on less than $1 per day by 2015, this is unlikely in many sub-Saharan African countries including Tanzania [1], which remains one of the poorest nations in the world, with a gross national income of $300 per capita in 2003 [2]. The Government of Tanzania has established key national economic policy frameworks [3,4], and has recognised the links between poverty and physical ill health, but the role of mental health had not been explicitly considered [5], despite the fact that in 1990 the World Bank estimated that neuropsychiatric disorders formed 10.5% of global burden of disease (DALYs) and suggested that this could rise to 15% in 2020. In fact they have already reached 19% [6]. They comprise 5 of the 10 leading causes of disability, and account for 28% of years of life lived with a disability. Depression alone is expected to rise from the fourth to the second leading cause of global disease burden as measured by DALYs by 2030. Depression contributes more than 10% of years of life lived with a disability, and suicide was the 10th leading cause of death. These figures do not take account of family burden or wider social costs.

The current study therefore aimed to examine the relationships in urban Tanzania between the rates of common mental disorder (CMD), such as depression and anxiety, and socio-economic factors, demographic characteristics and social functioning.

## 2. Methods

### 2.1. Sites

As previously described [7], in September and October 2003 a population-based survey was conducted in two urban areas of Dar es Salaam, Tanzania’s largest city with a population of 2.5 million. The areas were sites of the Adult Morbidity and Mortality Project (AMMP) [8,9], selected to ensure subpopulations of differing socio-economic circumstances. Ilala- Shaurimoyo (Ilala municipality) was a relatively middle-income area and Mtoni- Saba Saba (Temeke municipality) was a lower-income area [10].

### 2.2. Sample

A systematic random sample of 1,100 adults aged 15–59 was drawn from a random starting point from the entirely enumerated population of two geographically defined areas; 550 from the eligible population of 4,690 in Ilala-Shaurimoyo, and 550 from an eligible population of 11,620 in Mtoni-Saba Saba [11]. If the person randomly selected for interview in each household had moved away, the person who had moved into the house was interviewed instead. It was individuals rather than households which were enumerated, formed by the AMMP into a database of names, and hence sampled. Therefore, the sample was designed to be large enough in each area to provide estimates with adequate and similar levels of precision.

### 2.3. Procedure

The survey was coordinated by the Mental Health Section of the Ministry of Health, the Health Research Systems Section of the Directorate of Planning, and Dar es Salaam City Health Services. Interviewers were trained volunteer community health workers based in primary healthcare centres. Informed consent was obtained from all respondents.

### 2.4. Instruments

Information collected included demographic characteristics, socio-economic factors, recent life events and perceived social support. The Clinical Interview Schedule- Revised (CIS-R) [12], assessed common mental disorder, the Psychosis Screening Questionnaire (PSQ) [13], indicated the presence of psychotic symptoms, and the Alcohol Use Disorders Identification Test (AUDIT) [14], measured hazardous alcohol use. See related articles on psychotic symptoms [15], and alcohol use [7].

Demographic information collected included age, sex, ethnicity, marital status and household status (head, spouse or other). Socio-economic factors assessed included employment status, education attainment, income (whether or not the person had a cash income), housing tenure (owned or rented) and type of accommodation (whole house or room only).

The CIS-R [16], is a gold standard instrument for use by lay interviews in assessing psychopathology in community settings, which has been widely used in high [17–19], and low income countries [20–22], including Tanzania [23]. The CIS-R measures the presence of 14 symptom-types in the preceding month and if present, the frequency, duration and severity of each symptom in the past week. Scores taken together with algorithms based on the ICD-10 [24], provide diagnoses of depressive episode (mild, moderate or severe), obsessive compulsive disorder, panic disorder, phobic disorder, generalised anxiety disorder and mixed anxiety/depressive disorder. For the purpose of the current paper however, a score of 12 or more across the 14 sections of the survey was considered an indication of “any CMD”, as used in other CIS-R studies [12].

Respondents were given a list of 11 different stressful life events and asked to say which, if any, they had experienced in the last six months. The list included health risks (serious illness, injury or assault to self or close relative), loss of a loved one (death of a relative; death of a close friend), relationship difficulties (separation or divorce; serious problem with a close friend or relative); income instability (being made redundant or sacked; having looked for work for over a month; loss of the equivalent of three months income) and legal problems (problems with the police involving a court experience; something of value lost or stolen). The list was developed for the 1993 British psychiatric morbidity survey [17,25], further modified for the 1997 prisons survey [26], and slightly tailored for the Tanzania context. Scores were grouped into either “none”, “one”, “two” and “three or more” life events and were also analysed by category.

Perceived social support was assessed from respondents’ answers to seven questions which were used in the 1992 Health Survey for England [27], and the ONS Surveys of Psychiatric Morbidity [18,28]. The seven questions take the form of statements that individuals could say were not true, partly true or certainly true for them in response to the question ‘There are people I know who’; (i) Do things to make me happy; (ii) Who make me feel loved; (iii) Who can be relied on no matter what happens; (iv) Who would see that I am taken care of if I needed to be; (v) Who accept me just as I am; (vi) Who make me feel an important part of their lives; and (vii) Who give me support and encouragement. Results were categorised into no, moderate of sever lack of perceived social support.

Information on social networks was obtained through questions about the number of friends or relatives who informants felt close to, including (i) Adults who lived with the respondent and to whom they felt close; (ii) Relatives living elsewhere to whom they felt close; and (iii) Friends or acquaintances living elsewhere who informants would describe as close or good friends. These questions were taken from earlier psychiatric morbidity surveys [29,30], and results grouped “none to three”, “four to eight” and “nine or more”.

### 2.5. Analysis

Data were analysed using SPSS software for Windows Version 15 [31]. Chi squared (*χ*^2^) tests examined demographic and socio-economic differences between the areas, as well as differences in perceived social support and recent life events.

Odd ratios (ORs) with 95% confidence intervals (CIs) were calculated to determine significant associations with the presence of any CMD (*i.e.*, a CIS-R score of 12 more) and then “any CMD” was examined as the dependent variable in multivariate logistic regression. All factors significant in bivariate analyses and those that differed between areas plus age and gender were entered forward stepwise into the model. The 11 types of life event were collapsed into five categories and entered separately into the model to determine which categories of event were most strongly associated with CMD.

### 2.6. Ethics Approval

Approval was granted by National Institute for Medical Research, Ministry of Health, United Republic of Tanzania and South London and Maudsley (SLaM), National Health Service (NHS) Foundation Trust.

## 3. Results

### 3.1. Response Rate

The overall response rate was 82% with 899 out of 1,100 households approached completing the survey. Participation was higher in Ilala-Shaurimoyo where 481 out of 550 (87%) people agreed to complete the survey compared to Mtoni-Saba Saba households where 418 from 550 (76%) participated.

### 3.2. Differences between Areas

Living in poorer Saba Saba was associated with higher rates of CMD (4.1%) compared to the middle income area of Ilala Ilala (2.3%) although the difference was not statistically significant. Table 1 presents demographic and other characteristics by area. Respondents from Saba Saba and Shaurimoyo were of comparable age (p = 0.51), gender (p = 0.76) and marital status (p = 0.69) but respondents from Ilala were significantly more likely to be household head (p < 0.001), identify as non-African (p < 0.001) and to report renting their home (p = 0.04). Living in poorer Saba Saba was associated with unemployment (p < 0.001) and with younger school leaving age (p = 0.01). Saba Saba respondents also reported a significantly higher number of adverse life events in the six months preceding interview (P < 0.001).

### 3.3. Predictors of Common Mental Disorder

Table 2 shows the overall prevalence rates of any CMD by district and by gender. The overall prevalence of CMD was 31 per 1,000, and the prevalence was higher in women than in men in each district.

The prevalence was higher among females, people aged 16–24 and lowest among the married or cohabitating although these associations were not significant. Household status was significantly associated with CMD (p < 0.001) although given the very low prevalence among household heads (cell < 5), this finding should be considered with caution. The prevalence of any CMD was significantly higher among the unemployed compared to those with employment (p = 0.01) but there were no other significant associations with socio-economic factors. There was a strong and significant relationship between any CMD and number of recent life events (p < 0.001), with CMD was highest among those who reported three or more stressful experiences in the preceding six months.

There was no clear relationship between social support and CMD although rates were highest among those that perceived no lack of social support and people with large social support networks.

Factors significantly associated with any CMD (employment status and recent life events but not household status due to small cell size) as well as factors that varied significantly by area (ethnicity and housing tenure) were included in the multivariate logistic regression modelling. Gender and age included for a priori reasons. Number of recent life events was the only factor independently associated with CMD (Table 3).

Life events were also entered into the multivariate model by type of event rather than number of events again adjusting for factor associated with any CMD (employment status and recent life events), factors that varied significantly by area (ethnicity and housing tenure) and age and gender for a priroi reasons. Having experienced relationship difficulties, income instability and death of a loved one in the last six months remained independently associated with CMD (Table 4).

## 4. Discussion

### 4.1. Main Findings

To the best of our knowledge, this is the first community-based epidemiological study of common mental disorders in Tanzania, and also the first in Africa to extend the study of risk factors for CMD to household status, ethnicity, housing tenure, accommodation type, social support and area economic indicators. The main findings were that the 6 month prevalence of CMD was 41 per 1,000 and 23 per 1,000 in the respective study communities; and that across areas, CMD was independently associated with recently experiencing one or more stressful life events. There was no significant gender difference in prevalence of CMD.

### 4.2. Comparison with Other African Prevalence Studies

The prevalence rates found are relatively low compared to most other recent studies in sub Saharan Africa—see Table 5. An earlier review suggested the prevalence rate of CMD in Africa range between 8 and 43%, depending on the instrument used and population sampled [32]. Wide variation is also found in other regions of the world. The lifetime rate of any disorder across 17 countries was found to range between 12 and 47% [33].

However, past-year rates in Nigeria are comparable to our findings, where the prevalence of any anxiety and any mood disorder was 4.1% and 1.3% respectively [34]. Also consistent with the current findings, [35] in Nigeria reported an overall rate of depression of 5.2% across an urban and rural community.

### 4.3. Social Determinants of CMD

In the current study, the prevalence of CMD was higher among women although not significantly. Gureje and colleagues [34], in Nigeria also failed to demonstrate a difference between genders, and although consistent with Britain [36], and other parts of the world such as Brazil [37]. Higher rates among women have commonly been found in Africa including Zimbabwe [38], Ethiopia [39], and South Africa [40].

Older age (30+ years) was associated with lower odds of CMD, compared to people aged 16–24, whereas in Ethiopia rates were highest in people aged 35–44 [39], and 50–64 in Nigeria [34]. Patel and colleagues [41], in five low- and middle-income countries and Lima and colleagues [37], in Brazil also found higher rates in older age groups. In Britain, the peak age groups were 50–54 for men and 45–49 for women [19].

Marital status was not significantly associated with disorder although single, separated, divorced or widowed people had the highest rates of CMD. The lack of significant association is consistent with recent reports from Nigeria [34], and South Africa [40], although previous studies have identified marital status as a risk for CMD in developing countries (e.g., [21,42–45]), with higher rates in the widowed, separated and divorced reported in Pakistan [46], Ethiopia [39], and Brazil [37].

In our study of urban Tanzania, people who were not head of household had higher rates of CMD and few household heads reported symptoms. Similarly, a study in South Africa [47], found higher rates of illness in people with lower subjective social status and independent decision making.

Higher rates of disorder were found in non-Africans. In Britain rates were higher in South Asian and “other” groups than in white or black groups [36]. These findings support the view that ethnic minorities in a country have rates of illness which are both higher than indigenous populations, and higher than the populations of their former country [48]. On the other hand in South Africa, studies show higher rates in African rather than white populations, which probably reflects the socio-economic disparities of the two groups (e.g., Hamad *et al*. 2008 [47]).

There are previous African-based mental health surveys which have reported associations with socio-economic factors. A 2003 review identified 11 studies from developing countries that reported associations between CMD and indicators of poverty although only two were sub-Saharan African [49]. In the current study, rates of disorder were highest in people who left full time education between the ages of 14 and 16 compared to those who left at 13. In contrast, in surveys in Zimbabwe [41], Pakistan [46], Brazil [37], and Burundi [50], it was found that low education levels were strongly associated with CMD, and indeed British rates of CMD were higher in those who leave school before 16 compared to those who completed school [28].

In Tanzania, as in Britain [36], the unemployed had significantly higher rates of disorder although the association did not remain after adjustment for other factors. Similarly in Zimbabwe, Patel [41], found the association with unemployment was not consistent after adjustment for age and sex.

Income was not significantly associated with CMD although higher rates of disorder were among those with an income. This surprising finding however is consistent with Gureje and colleagues [34], who found having an income to significantly increase the risk of any disorder, suggesting that receipt of a monetary income may bring with it additional life stress, possibly caused by the social demands and responsibilities experienced by those with an income.

Two urban areas of differing socio-economic circumstances were surveyed in this study, Median estimated household monthly income in 2002 was 17,752 TZS ($US 20.90) in Mtoni-Saba Saba, significantly lower than in Ilala 22,308 TZS ($US 26.26) [51]. Living in the less affluent area of Mtoni Saba Saba was associated with higher rates of disorder (4.1%) compared to the middle-income area of Ilala Ilala (2.3%) although the difference was not statistically significant.

### 4.4. Life Events and CMD

There was a strong positive association between life events and CMD in urban Tanzania, where the odds of experiencing CMD were far greater for those who had experienced three or more in the preceding six months. Tafari and colleagues [39], in Ethiopia found that people who had experienced six or more stressful life events in the past year had 2.7 times greater odds of neurosis. A strong association between life events and depression has been reported in Zimbabwe [52], and Pakistan [46]. The relationship between life events and CMDs has been demonstrated elsewhere including Brazil [37], and is well established in Britain [53]. It has been suggested that in addition to key demographic characteristics, life events have greater influence on mental health than poverty *per se* in the developing context [54].

### 4.5. Social Support and CMD

Those with no social support were significantly more likely to have CMD in the UK [28], while the direction of the association was opposite in Tanzania, with the rate of CMD increasing with increasing amount of perceived support and size of support group. A study in South Africa [55], found no independent association between levels of social support and psychological distress, while a Zimbabwean study found crisis support from family members to be protective [56]. However, a UK mental health survey of different ethnic groups found that high CIS-R scores (indicating presence of CMD) was associated with higher number of friends and relatives among Bangladeshi and Indian respondents, but not the other ethnic groups in the survey [57]. Further investigation is warranted in Tanzania. It may be that those with a large social network found that the support which may be derived from the group was outweighed by the heavy familial demands made by large extended families for financial and practical support.

Few African-based mental health surveys have reported associations with socio-economic factors. A 2003 review identified 11 studies from developing countries that reported associations between CMD and indicators of poverty although only two were sub-Saharan African [49]. In the current study, rates of disorder were highest in people who left full time education between the ages of 14 and 16 compared to those who left at 13. In contrast, surveys in Zimbabwe [43], Pakistan [34], Brazil [35], and Burundi [50], it was found that low education levels were strongly associated with CMD. British rates were higher in those who leave school before 16 compared to those who completed school [27].

### 4.6. Limitations of the Study

The sampling frame was well-defined and based on bi-annual demographic surveillance for the AMMP. However, current findings are specific to the two wards in urban Dar es Salaam and are not necessarily applicable to other parts of Tanzania, particularly rural areas. The combination of pencil and paper administration of the survey and the complex routing of the CIS-R resulted in missing data and possible underestimation of prevalence rates. In the regression analysis, data from the two areas were combined without weighting for the different probabilities of selection although variables that differed significantly between areas were included in the logistic regression modelling.

## 5. Conclusions

This study of two urban areas of Tanzania of differing levels of poverty did not find a significant difference in rates of CMD between areas although the poorest area did have higher rates of disorder. CMD is highly associated with exposure to traumatic life events in urban Dar es Salaam, particularly events involving relationship difficulties and financial instability. The effect of social support was counter intuitive and deserves further research. Efforts to address poverty and disadvantage in low income countries such as Tanzania will need to take mental health into account and address the difficult circumstances and environments within which people live and work.

## Figures and Tables

**Table 1.** Demographic & socio-economic factors, social support and life events by area.

	**Saba Saba n = 418 (%)**	**Ilala n = 481 (%)**	**Unadjusted odds ratio (95% CI)**	**P value (χ^2^)**
**Gender**				
Male	44	43	1.00	
Female	56	57	1.04 (0.80–1.36)	0.760
**Age**				
16–24	32	29	1.00	
25–34	34	34	1.11 (0.80–1.54)	
35+	34	37	1.21 (0.88–1.68)	0.508
**Marital status**				
Married/cohabitating	55	56	1.00	
Single	38	36	0.93 (0.71–1.23)	
Widowed/divorced/separated	8	9	1.16 (0.71–1.89)	0.687
**Relationship to household head**				
Head	35	45	1.00	
Spouse/partner	32	33	0.81 (0.59–1.11)	
Other	34	23	0.52 (0.38–0.73)	<0.001
**Ethnic group**				
Black African	98	88	1.00	
Other	2	12	7.6 (3.47–17.09)	<0.001
**Employment status**				
Working	32	39	1.00	
Unemployed	9	3	0.26 (0.13–0.51)	
Economically inactive	59	58	0.81 (0.60–1.08)	<0.001
**Own/rent accommodation**				
Owns	49	41	1.00	
Rents	48	55	1.35 (1.03–1.77)	
Rent free	2	4	1.99 (0.90–4.38)	0.036
**Type of accommodation**				
Whole house	46	41	1.00	
Rooms/flat/other	54	59	1.24 (0.95–1.62)	0.107
**Age left full time education**				
13 or under/Never went	8	5	1.00	
14–16	38	38	1.69 (0.94–3.07)	
17 or 18	24	24	1.69 (0.91–3.12)	
19+ yrs	20	27	2.32 (1.25–4.30)	
Still at school	10	6	1.02 (0.49–2.11)	0.012
**Income**				
Yes	44	41	1.00	
No	55	59	1.14 (0.87–1.50)	0.349
**Perceived social support**				
Severe lack	21	21	1.00	
Moderate lack	37	34	0.94 (0.65–1.38)	
No lack	41	44	1.08 (0.75–1.56)	0.708
**Size of Primary Social support group**				
0–3	14	15	1.00	
4–8	42	49	1.12 (0.75–1.67)	
9 or more	44	36	0.79 (0.53–1.18)	0.408
**Number of life events**				
None	56	72	1.00	
1	26	21	0.61 (0.44–0.84)	
2	12	6	0.37 (0.23–0.61)	
3+	7	3	0.28 (0.14–0.56)	<0.001

**Table 2.** Prevalence rates of any CMD by districts and gender.

Prevalence (Per 1,000 in last 6 months)	Temeke	Ilala	Combined
Men	38	14	25
Women	43	29	36
All Adults	41	23	31

Data from the two areas were combined to investigate correlates of any CMD (see Table 3).

**Table 3.** Unadjusted and adjusted odds ratios for demographic and socio-economic variables and common mental disorder.

	**Sample size**	**Prevalence of CMD (%)**	**Unadjusted odds ratio (95% CI)**	**Adjusted odds ratio (95% CI)**
**Gender**				
Male	393	2.5	1.00	
Female	506	3.6	1.41 (0.65–3.10)	
**Age**				
16–24	275	4.7	1.00	
25–34	308	3.2	0.68 (0.29–1.57)	
35+	316	1.6	0.32 (0.11–0.92)	
**Marital Status**				
Married/cohabitating	495	2.4	1.00	
Single	327	4.0	1.67 (0.75–3.70)	
Widowed/divorced/separated	75	4.0	1.68 (0.46–6.09)	
**Relationship to household head**				
Head	359	0.6	1.00	
Spouse/partner	290	4.5	8.38 (1.88–37.43)*	
Other	250	5.2	9.79 (2.19–43.78)*	
**Ethnic group**				
Black African	834	3.1	1.00	
Other	63	3.2	1.02 (0.24–4.39)	
**Employment status**				
Working	300	1.7	1.00	
Unemployed	49	10.2	6.71 (1.87–24.10)*	
Economically inactive	496	3.6	2.22 (0.82–6.05)	
**Own/rent accommodation**				
Owns	403	4.0	1.00	
Rents	463	2.6	0.64 (0.30–1.38)	
Rent free	29	0.0	–	
**Type of accommodation**				
Whole house	386	3.4	1.00	
Rooms/flat/other	510	2.9	0.87 (0.41–1.85)	
**Age left full time education**				
13 or under/Never went	52	1.9	1.00	
14–16	337	3.0	1.56 (0.20–12.44)	
17+ years	420	2.9	1.5 (0.91–11.78)	
**Income**				
Yes	354	4.5	1.00	
No	476	2.3	0.50 (0.23–1.09)	
**Perceived social support**				
Severe lack	173	2.1	1.00	
Moderate lack	288	3.1	1.36 (0.41–4.49)	
No lack	341	4.1	1.81 (0.59–5.58)	
**Size of Primary Social support group**				
0–3	130	1.5	1.00	
4–8	411	2.9	1.92 (0.43–8.71)	
9 or more	358	3.9	2.6 (0.58–11.62)	
**Number of life events**				
None	576	0.7	1.00	1.00
1	206	4.9	7.30 (2.26–23.53)*	7.22 (2.24–23.28)*
2	46	6.6	10.07 (2.64–38.37)*	9.91 (2.60–37.80)*
3+	41	22.0	40.22 (11.75–137.66)**	39.83 (11.06–136.78)**

* p < 0.01,

** p < 0.001.

**Table 4.** Odds ratios for different categories of life events and mental disorder.

	**Sample size**	**Prevalence of CMD (%)**	**Unadjusted odds ratio (95% CI)**	**Adjusted odds ratio^a^(95% CI)**
**Health risks**				
No	815	2.7	1.00	
Yes	84	7.1	2.77 (1.09–7.04)*	
**Relationship problems**				
No	851	2.1	1.00	1.00
Yes	48	20.8	12.18 (5.27–28.17)***	8.39 (3.39–20.76)***
**Income instability**				
No	805	1.7	1.00	1.00
Yes	94	14.9	9.89 (4.55–21.48)***	6.63 (2.91–15.12)***
**Death of a loved one**				
No	702	2.1	1.00	1.00
Yes	197	6.6	3.24 (1.51–6.92)**	2.31 (1.01–5.25)*
**Legal issues**				
No	859	2.7	1.00	
Yes	40	12.5	5.19 (1.86–14.47)**	

a Adjusted for age, sex, employment status, ethnicity, housing tenure and other event types;

* p < 0.05;

* p < 0.01;

*** p < 0.001.

**Table 5.** Prevalence studies of common mental disorders in sub Saharan Africa.

**Country**	**Author**	**Setting**	**Number**	**Measure**	**Prevalence**
Lesotho	Hollifield *et al.* 1990	Rural	356	DIS	23.5% F 14.7% M
Zimbabawe	Abas & Broadhead, 1997	Urban	172	SSQ	15.7% F 1 month, 30.8% F 1 year
South Africa	Haavenaar *et al.* 2008	Periurban	209	SRQ	34.9% M and F
South Africa	Haavenaar *et al.* 2008	Rural	222	SRQ	27.0% M and F
South Africa	Hamad *et al.* 2008	Urban	257	CES-D	64.5% F, 50.4 % M
Nigeria	Gureje *et al.* 2006	Probability sample of 8 states	4,984	CIDI	12.1% life time; 5.8% 12 month prevalence M and F
Ethiopia	Tafari *et al.* 1991	Rural	2,000	SRQ	11.2% M and F
Uganda	Orley and Wing 1979	Rural		PSE	
Sudan	Rahim and Cederblad	Urban		SRQ/DSM III	16.6% M and F
